# Antitumor Virotherapy by Attenuated Measles Virus (MV)

**DOI:** 10.3390/biology2020587

**Published:** 2013-03-28

**Authors:** Jean-Baptiste Guillerme, Marc Gregoire, Frédéric Tangy, Jean-François Fonteneau

**Affiliations:** 1 INSERM, UMR892, Nantes, F-44000, France; E-Mails: jean-baptiste.guillerme@univ-nantes.fr (J.-B.G.); marc.gregoire@nantes.inserm.fr (M.G.); 2 CNRS, UMR6299, Nantes, F-44000, France; 3 Université de Nantes, Nantes, F-44000, France; 4 CNRS, URA3015, Institut Pasteur, Unité de Génomique Virale et Vaccination, Paris, 75015, France; E-Mail: ftangy@pasteur.fr

**Keywords:** antitumor virotherapy, measles virus vaccine, dendritic cells, tumor antigen, clinical trial, vaccine

## Abstract

Antitumor virotherapy consists of the use of replication-competent viruses to infect and kill tumor cells preferentially, without damaging healthy cells. Vaccine-attenuated strains of measles virus (MV) are good candidates for this approach. Attenuated MV uses the CD46 molecule as a major entry receptor into cells. This molecule negatively regulates the complement system and is frequently overexpressed by cancer cells to escape lysis by the complement system. MV exhibits oncolytic properties in many cancer types *in vitro*, and in mouse models. Phase I clinical trials using MV are currently underway. Here, we review the state of this therapeutic approach, with a focus on the effects of MV on the antitumor immune response.

## 1. Introduction

During the past decade, there has been an increasing interest in antitumor virotherapy using oncolytic viruses (OV). This consists of the use of replication-competent virus to infect and kill tumor cells preferentially, without, or with limited, infection of healthy counterparts. The oncolytic properties of many live, attenuated viruses have been evaluated as cancer therapeutics, including adenovirus, vesicular stomatitis virus, herpes simplex virus, Newcastle disease virus and MV [1]. The tropism of OV for tumor cells is due to two main factors. First, the receptor that OV use to enter cells is overexpressed by, or has expression restricted to, tumor cells. The second level of selectivity is tumor biology [2]. Most tumors develop many strategies to escape the immune system, and to resist apoptosis and translational suppression. These mechanisms also play crucial roles in the control of viral replication. Their ablations during tumor development make the tumor an efficient destination for virus to replicate. Tumor cell death through OV infection is complex and should be considered as a particular/original type of cell death which differs from classical cell death by apoptosis, necrosis or autophagy [2].

In addition to killing tumor cells, OV could play a crucial role in tumor rejection to overcome or reorient immune suppression mediated by the tumor microenvironment. By its nature, the virus is a “danger signal” able to recruit immune cells such as dendritic cells (DC) [3]. DC are at the interface between innate and adaptive immunity, and can prime an adaptive immune response, notably T cell responses against specific, tumor-associated antigens if these are correctly activated [4].

Among the different oncolytic viruses, vaccine-attenuated strains of measles virus (MV) show a promising potential for cancer therapy. As early as the 1970s, spontaneous clinical remissions were observed in patients suffering from hematological cancers after natural infection with wild-type (wt) MV [5,6]. Since then, it has been demonstrated that attenuated MV exhibits oncolytic properties against many types of cancers, *in vitro* and *in vivo*, in models of human tumor xenografts in immunodeficient mice [1]. Alternatively, the capacity of MV infection to activate a strong immune reaction could play a crucial role in the efficiency of antitumor virotherapy based on MV [7,8]. Antitumor virotherapy using MV is, thus, currently being evaluated in clinical trials [2].

Oncolytic virotherapy is a complex approach, requiring good viral replication in targeted tumor tissue and the enrollment of the immune system against tumor-associated antigens, without inhibiting earlier viral replication. Here, we review the different aspects of this approach using attenuated strains of MV, with a special focus on MV immunogenicity and effects on the antitumor immune response.

## 2. MV Vaccine

### 2.1. Measles Virus Description: wt and Vaccine-Attenuated Strains

MV is an enveloped and negative, nonsegmented, single-strand RNA virus belonging to the *Morbillivirus* genus of the *Paramyxoviridae* family [9]. The World Health Organization lists twenty-four strains of measles viruses, classed into eight clades [10]. The MV RNA genome comprises around 16,000 nucleotides and encodes eight proteins, two of which are nonstructural proteins (V and C) resulting from an alternative transcript from the RNA coding for the phosphoprotein (P). Large protein (L) and nucleoprotein (N) form the viral nucleocapsid, which contains the viral RNA genome. The matrix protein (M), fusion protein (F) and hemagglutinin protein (H) form the viral envelope with lipids from the infected host cell membrane [9].

The first MV isolate was obtained from a child and was named the Edmonston strain. Different attenuated strains of MV have been generated from the wild-type Edmonston strain by culture in various cell types [11,12]. For example the Schwarz strain, which is produced and used to vaccinate children in Europe and Brazil, was generated in 1962 by additional subcultures of the Edmonston strain in chick embryo fibroblasts [13]. The comparative sequence analysis between wild-type-Edmonston and Schwarz shows 42 nucleotide changes. These mutations contribute to the attenuated profile of this strain [12].

The replication cycle starts with the adsorption of MV to the host cell through the interaction between the MV H protein and the cell surface molecules CD150, CD46 and/or Nectin-4. The F protein then mediates the fusion between the viral particle and the host cell membrane, allowing penetration of the negative, single-stranded RNA and the associated proteins into the cytoplasm. These proteins form a ribo-nucleo-proteic complex (RNP complex), which is a template for mRNA transcription and MV genome replication [12]. The virions bud from the plasma membrane of the host cell to end the cycle. In addition, MV infection is known to induce the formation of syncytia. MV-infected cells fuse with uninfected neighboring cells forming multinucleated infected cells, a phenomenon that increases the efficiency of MV replication.

### 2.2. Vaccine Efficacy

Measles virus is a highly contagious agent and one of the leading causes of death in children worldwide [9]. One person with measles can infect fifteen to twenty others (R_0_ = 15–20), meaning that the interruption of endemic transmission in a population requires that more than 95% of the population is immune [14]. People who recover from measles are immunized for life. Most measles-related deaths are caused by MV-associated temporary immune suppression which allows opportunistic infections. Even though a safe and cost-effective vaccine is available, around twenty million cases of measles infection are reported every year, and more than 95% of MV-related deaths occur in underdeveloped countries [15].

Live, attenuated MV vaccine has been distributed at low cost to millions of children in many countries through the Extended Program on Immunization (EPI) of the World Health Organization (WHO). This vaccine induces a strong, long term immunity against measles virus after one or two injections, as antibodies and specific CD8+ T cells persist for as long as 25 years after vaccination [16]. Prior to vaccination, an estimated four to five million cases of measles occurred annually between 1953 and 1962 in the US [17]. From 2001 to 2008 the annual incidence of measles was less than one case per million in the US [18], and less than ten cases per million in Europe [19]. Measles caused an estimated 139,300 deaths worldwide in 2010, which represents a greater than 97% decrease compared with the estimated six million deaths occurring annually prior to use of the measles vaccine [20].

### 2.3. Vaccine Safety: Absence of Observed Reversion

From 1978 to 2010, 17,536 adverse experiences following immunization were reported to Merk after M-M-R^TM^ II administration, for approximately 575 million doses distributed. Only 4,822 cases were considered to be serious, the relative risk of a serious event thus being 8.4 cases per million doses distributed [17]. The reversion of measles vaccine to a pathogenic form has never been reported [21]. Altogether, these considerations make the measles virus vaccine one of the safest human vaccines.

## 3. MV in Antitumor Virotherapy

### 3.1. Receptors for MV Infection

Receptors for the entry of MV are quite well defined. The pathogenic wild-type strains of measles virus enter the cell using the signaling lymphocyte activation molecule (SLAM/CD150) which confers on this virus a natural tropism for T and B lymphocytes and activated monocytes/macrophages [22,23]. The live, attenuated strains of MV, which are selectively oncolytic, use the CD46 molecule as the major receptor for entry into the cell [24,25,26], which is ubiquitously expressed at a low level by all nucleated cells [27]. CD46 and other complement regulatory proteins (CRP: CD55 and CD35) block the complement cascade at the C3 activation stage, and their expression at low density protects normal tissues from accidental injury by activated complement. It is also clear that many tumor types overexpress these molecules to escape complement-dependent cytotoxicity (CDC) [28,29]. This selective expression at high density by many cancer cell types confers on live, attenuated MV a natural tropism for tumor cells. Indeed, it has been demonstrated that, above a certain threshold of CD46 expression, the killing and syncitia formation mediated by MV infection increase dramatically [24] whereas healthy tissues, which express a low density of CD46, remain unharmed [30].

Nectin-4 (PVRL-4) has recently been identified as a novel receptor for wtMV and vaccine strains [31,32] and plays a crucial role in the shedding of MV from the respiratory tract of infected individuals for transmission of the disease [33]. It is an adherent junction protein originally described as a member of the polio virus receptor-like proteins, which act as an entry site for poliovirus [34] and other viruses such as HSV-1 and HSV-2 [35]. In humans, PVRL-4 is mostly expressed in placenta and trachea, and can also be found at a low density in the epithelial cells of tonsils, oral mucosa, lung macrophages and neuronal cells of the cerebral cortex [31]. It has also been described as a tumor marker which is frequently overexpressed in many adenocarcinomas, such as ovarian, lung, colon and breast tumors [36,37,38]. It has been demonstrated recently that nectin-4 is necessary for the tumor selectivity of infection by MV in some breast cancers [39].

Finally, other MV entry receptors may exist, as some tumor cell lines which do not express CD46, CD150/SLAM and nectin-4 are sensitive to MV infection [40].

### 3.2. MV Sensitivity of Numerous Cancer Types *In Vitro* and *In Vivo*

The cytopathic effect (CPE) of MV is characterized by the formation of syncytia (giant multinucleated cells) due to the interactions of viral hemagglutinin and fusion proteins from the infected cells with the CD46 expressed by the neighboring cells. Many studies have described oncolytic properties of MV, *in vitro* and *in vivo*, on immunodeficient mice bearing human tumor xenografts. Hence, oncolytic efficacy of MV has been demonstrated against T-cell lymphoma [41,42], myelomas [43], pancreatic cancer [44], glioblastomas, gliomas [45,46], ovarian carcinomas [30], prostate cancer [47], breast cancer [39,47,48], melanoma [49], renal cell carcinoma [50], mesothelioma [7,51], medulloblastoma [52,53] and lung/colorectal adenocarcinoma [54]. Recently, Zhang *et al.* demonstrated a therapeutic efficacy of MV against human hepatoblastoma, both *in vivo* and *in vitro* [55]. During the last ten years, oncolytic activities of MV have been evaluated and demonstrated for at least twelve different cancer types, highlighting the enthusiasm given to this approach. Furthermore, the fact that attenuated MV has been injected to vaccinate millions of children around the world with an excellent safety profile and with no observed reversion to the wt-MV make MV attractive, compared to other OV that are not used in vaccination.

### 3.3. Engineering of MV to Increase the Efficiency of Antitumor Virotherapy

Despite its natural tropism for tumor cells and its lytic activity, the oncolytic properties of MV can be improved by genetic engineering. RNA encoding recombinant protein can be inserted into the MV, up to 5 kb [56]. Thus, recombinant MV can be designed to express receptors against specific tumor markers, to increase its replication capacity, or to improve the monitoring of viral spread *in vivo*.

Virus entry into cells is dependent on cell-surface-marker recognition and the concept of engineering MV to extend its recognition of tumor-specific markers is interesting. Hence, the group of M.S. Topp efficiently retargeted MV for multiple myeloma (MM). Using site-specific mutagenesis, they inserted the sequence encoding a single chain derived from the Wue-1 antibody, which specifically recognizes CD138+ MM [57]. This modification makes the MV unable to infect cells by CD150 or CD46. Despite the lack of classical recognition, the retargeted MV-Wue has shown efficient oncolytic properties against MM [58].

Another way to improve the oncolytic potential of MV is to improve its cytotoxic activity. Meng and colleagues developed an MV derived from Edmonston lineage, recombinant for wild-type N/P/L protein (MV-NPL). These proteins form the ribonucleoprotein complex which is the viral replication unit in infected cells. These genetic modifications confer on MV more efficient replication and cytotoxic abilities against renal cancer cells than nonengineered MV, and display a minimal cytopathic effect on normal human cell lines [50].

MV has also been engineered to facilitate the monitoring of viral spread *in vivo*. E. Galanis’ group has developed an MV recombinant for carcinoembryonic antigen (CEA). CEA is used as a reporter gene for viral replication, which can be detected in the blood. This MV-CEA exhibits efficacy against different types of tumor models, *in vitro* and *in vivo* [46,59], and has also been tested in a phase I clinical trial in recurrent ovarian cancer patients, by intraperitoneal injection [60].

MV has also been genetically modified to express the sodium iodide symporter, NIS, (MV-NIS). This modification allows the noninvasive assessment of MV replication by administration of ^123^I for gamma-camera imaging, as infected tumor cells express NIS making them permeable to ^123^I. MV-NIS was also used to improve the tumoricidal properties of MV, by allowing an ionizing radiation treatment by administration of ^131^I, a β emitter. The β particles penetrate the tissue for 0.4 mm and provide a local bystander killing of oncolysis-refractory tumor cells [61].

Type I IFNs are known to have antiangiogenic properties [62] and to induce an antitumor immune response by modulating the activation of different immune cell types, such as NK and T cells [63]. These properties have been exploited in an engineered MV, modified in order to express recombinant INF-β. Li and colleagues demonstrated that MV-IFN-β displayed a better capacity to kill mesothelioma xenografts and to increase the survival of treated mice than mice treated with an MV not recombinant for IFN-β [51]. This study also revealed that MV-IFN-β allows a modification of the tumor microenvironment, by increasing the infiltration of CD68+ macrophages and limiting neoangiogenesis.

Recently, a Schwartz attenuated strain of MV containing a transgene encoding the suicide gene super cytosine deaminase (MV-SCD) has been studied to kill sarcoma tumor cell lines that are sensitive to MV infection, but where the MV fail to replicate due to innate antiviral mechanism, such as IFN-stimulated genes [64]. SCD is a gene encoding a fusion protein consisting of yeast cytosine deaminase and yeast uracil phosphoribosyltransferase that transforms the prodrug 5-fluorocytosine into a cytotoxic drug.

## 4. MV and the Antitumor Immune Response

### 4.1. MV Immunogenicity May Participate in the Efficiency of Antitumor Virotherapy

In addition to its capacity to lyse infected tumor cells, MV has also the ability to activate the immune system. This ability may participate in the efficiency of antitumor virotherapy based on MV. Indeed, tumors develop many different strategies to grow, especially by conditioning their microenvironment to escape the immune system and to promote neoangiogenesis. They produce immunosuppressive cytokines, notably TGF-β, and recruit immunosuppressive cells, such as T regulatory cells (Tregs), to maintain immune tolerance within the tumor. Thus, MV may also act as an immunoadjuvant to overcome the immunosuppressive tumor environment. It has already been demonstrated for reovirus, another OV, that innate and adaptive immunity-mediated antitumor activity is necessary for successful virotherapy, independent of direct oncolysis [65,66]. Infection at the tumor site can induce a potent immune response due to the release of “danger signals” induced by MV-mediated cell death. These danger signals can be classified into two categories: the damage-associated molecular patterns (DAMPs) which are released as a result of immunogenic cell death, notably triggered during viral infection [67], and the pathogen-associated molecular patterns (PAMPs) which are conserved molecular motifs expressed by pathogens such as the single stranded RNA of MV [68]. The presence at the tumor site of danger signals (DAMPs and PAMPs) and dead tumor cells as a source of tumor antigens may fulfill the conditions for an efficient induction of antitumor immunity.

### 4.2. MV-Infected Tumor Cells Activate Myeloid Dendritic Cells

In humans, there are at least two main subsets of DC: the CD11c+/BDCA2- myeloid DC (mDC), which include Langerhans cells and dermal and intestinal DC, and the CD11c-/BDCA2+ plasmacytoid DC (pDC). Each subset is equipped with a specialized combination of pattern-recognition receptors (PRR), allowing each DC subset to recognize different danger signals.

mDC constitute a major antigen-presenting cell family and play a crucial role in the induction of tolerance or immune response. In normal conditions they tend to induce tolerance against self antigens, whereas in pathogenic conditions the presence of danger signals induces their maturation, notably their expression of costimulatory molecules (CD80, CD86), and allows them to prime T cell responses [4,69]. DC are defined by a typical morphology [70] and a unique ability to acquire and process exogenous antigen into peptides for class II major histocompatibility complex (MHC) presentation to CD4+ T cells in lymphoid organs, and also for class I MHC presentation to CD8+ T cells, a phenomenon named “cross-presentation” [71]. Their maturation/activation is required to prime adaptive immunity [72]. Recruitment of DCs could be a key regulator in the long term therapeutic benefit of oncolytic therapy by triggering an antitumor immune response, notably T cell responses. Therefore, stimulation of the mDC subset to promote tumor rejection is probably essential to the efficacy of antitumor virotherapy.

The effects of MV-infected tumor cells on mDC have been addressed recently. Our group demonstrated, *in vitro*, that mesothelioma tumor cells infected with the Schwarz strain of MV undergo immunogenic cell death characterized by the release of DAMPs, such as the heat shock proteins, Hsp70 and gp96, and by the presence of the viral double-stranded RNA (dsRNA) [7]. Monocyte derived-DC mature after internalization of MV-infected tumor cells, due to their expression pattern of PRR (TLR3, MDA5, RIG-1) and the presence of DAMPs and PAMPs. This efficient uptake and maturation allows DC to cross-present tumor antigen, and, thus, to cross-prime CD8+ T cells specific for mesothelin, a tumor antigen expressed by mesothelioma tumor cells [7]. Donnelly and colleagues also demonstrated that MV Edmonston strain induced immunogenic death of melanoma tumor cells, through the secretion of inflammatory cytokines (IL-6, IL-8 and type I IFN) and chemokines (RANTES and HMGB1) in an MV dose-dependent manner [49]. They also show that MV-infected tumor cells are a good substrate to induce an antitumor T cell response by cross-presentation. The release of HMGB1 by MV-infected tumor cells is of particular interest as it has been shown to be a potent DAMP important for immunogenic cell death of cancer cells, acting as an adjuvant to DC maturation through the TLR4 pathway [73].

Recently, a new subset of mDC that is more efficient than the others to perform cross-presentation, has been described [74,75,76,77]. This new DC subset is characterized by expression of BDCA-3 membrane protein (CD141), the chimiokine receptor XCR1, and the C-type lectin Clec9A (DNGR-1). Interaction of this new subset of mDC with MV has not been addressed.

### 4.3. MV-Infected Tumor Cells Activate Plasmacytoid DC (pDC)

pDC, also known as type I interferon-producing cells (IPC), are present in the blood and lymphoid organs. This subset of DC is particularly involved in the antiviral immune response, due to their expression of intracellular toll-like receptors specialized in sensing viral nucleic acid (TLR7 and 9). Hence, pDC respond to a wide range of RNA and DNA viruses, in response to TLR triggering, by producing large amounts of type I IFN (IFN-α, -β) and by upregulating their expression of maturation markers and costimulation molecules. pDC have already shown their capacity to present viral antigens to CD8+ and CD4+ T cells when they are infected by influenza virus [78], and to cross-present viral antigen acquired from infected cells to CD8+ T lymphocytes [79,80].

We recently demonstrated that MV antitumor virotherapy could be an efficient way to recruit pDC in antitumor immunity [8]. We showed, *in vitro*, that MV-infected melanoma cell lines are efficiently internalized by pDC and induce their maturation, characterized by a huge secretion of IFN-α in a TLR7-dependent manner. We demonstrated that human pDC can cross-present a tumor-associated antigen (NYESO-1) to CD8+ T cells from the MV-infected tumor cells. We also observed that cross-presentation occured only when pDC acquired antigen from MV-infected tumor cells, whereas pDC exposed to UV-irradiated tumor cells (nonimmunogenic cell death) did not perform tumor antigen cross-presentation. These results show that MV-infected tumor cells activate pDC, notably their capacities to produce large amounts of IFN-α and to cross-present tumor antigens to CD8+ T cells. IFN-α plays an important role in the antitumor immune response, as it is necessary for the induction of tumor-antigen-specific T cell responses [81,82].

pDC have been shown to play a beneficial role in the antitumor immune response, when they are adequately activated. It has been demonstrated recently, in a mouse melanoma model, that topical imiquimod treatment of tumors can induce tumor rejection. Imiquimod is a ligand of TLR7, expressed by pDC, and activates their cytotoxic properties through the expression of TRAIL and the secretion of granzyme B [83]. In a similar model, C. Liu *et al.* show that injection of TLR9-activated pDC induces tumor regression in mice by recruitment of NK cells at the tumor site in a CCR5-dependent manner [84]. Furthermore, J. Mourries *et al.* have shown in a mouse model that antigen cross-presentation by pDC can be triggered by viruses or by TLR7 or TLR9 ligands, which results in cross-priming of antigen-specific CD8+ T cells [85]. Finally, Jolanda De Vries group has recently completed the first phase I clinical trial by intranodal injection of tumor-antigen-loaded and -activated pDC in metastatic melanoma patients. They reported that all patients mounted a T cell response against the tumor antigens. The clinical outcomes of this trial were promising with a significantly extended survival in the majority of the patients [86].

Thus, MV used in antitumor virotherapy may be an interesting method of activating pDC subsets in cancer patients. Furthermore, it has an additional advantage over classical TLR7 or TLR9 ligands such as imiquimod, as MV induces the release of tumor antigens for cross-presentation following the lysis of infected tumor cells.

### 4.4. Effects of MV on other Immune Cell Types

Besides DC, the effects of MV virotherapy on the antitumor immune response have also been studied on neutrophils [87]. In a human lymphoma xenograft model in immunodeficient mice, intratumoral injection of MV induced the regression of large tumors, characterized by an important influx of activated neutrophils in the tumor. The number of these neutrophils correlated with the level of tumor regression, suggesting a role for these cells in the efficiency of antitumor virotherapy by MV.

It is also possible that MV virotherapy is able to induce natural killer (NK) cell activation, as IFN-α produced by pDC is able to activate an NK response [84]. However, D. Grote *et al.* failed to observe an influx of NK into tumor in their model of MV virotherapy using a human lymphoma xenograft in immunodeficient SCID mice [87]. The authors did not look at cells of the adaptive immune response, since T and B lymphocytes are deficient in SCID mice.

This last study highlights one problem encountered by immunologists to determine the effect of MV virotherapy on antitumor immune response. Indeed, mouse cells, including tumor cells, are not sensitive to MV infection. Thus, this therapeutic approach cannot be studied in immunocompetent mice. Most *in vivo* work on MV virotherapy has been performed with human tumor xenografts in immunodeficient mice (nude, SCID, Nod/SCID), which limits study of the antitumor immune response on some cells of the innate immune response, such as neutrophils or NK, but does not allow to study the adaptive antitumor immune response. This is not the case of some other OV, such as vesicular stomatitis virus or vaccinia, which can infect human cells, as well as mouse cells [2].

### 4.5. Effects of Anti-Measles Immune Response on MV Antitumor Virotherapy

Due to the very high contagiousness of wt-MV and to MV vaccination programs started at the end of the 70s, more than 90% of occidental countries population is immunized again MV, with notably presence of neutralizing antibodies that can persist throughout life [16]. These neutralizing antibodies may limit infection of tumor cells by MV. Indeed, in a model of human disseminated myeloma xenograft in immunodeficient mouse, the presence of adoptively transferred neutralizing antibody impaired efficiency of MV antitumor virotherapy when the MV is injected systematically [88]. One way to circumvent this problem is to inject MV-infected cells as virus carriers, which protect the virus from neutralizing antibodies, instead of injection of free MV. This virus carrier strategy has also been used to treat orthotopic ovarian cancer by intraperitoneal injection of mesenchymal stem cells infected with MV [89]. Thus, systemic injection of free MV for antitumor virotherapy is not efficient due to the presence of neutralizing antibodies. Virus carrier is a strategy that permits to limit effects of neutralizing antibody. 

Another approach to limit effects of neutralizing antibodies may be to inject MV directly inside tumors. In a recent phase II clinical study using as OV, vaccinia virus with disruption of the viral thymidine kinase gene and engineered to express GM-CSF to treat advanced hepatocellular carcinoma, Heo *et al*. show that intratumoral injections of high doses of virus induce strong clinical responses which are independent of a preexisting immunity against vaccinia in treated patients [90].

## 5. Clinical Trials

### 5.1. Phase I Clinical Trial on Cutaneous T Cell Lymphoma (CTCL)

Viral oncolysis has been proposed as a mechanism explaining the spontaneous tumor regressions of Hodgkin lymphoma [91,92] and Burkitt’s lymphoma [5,93] after natural MV infections. These case reports suggest an important oncolytic activity for MV against hematological tumors. These observations led Heinzerling and colleagues to carry out the first phase I clinical trial of MV antitumor virotherapy using the Edmonston-Zagreb strain of MV on patients with CTCL [94]. This malignancy is characterized by an accumulation of clonal T lymphocytes in the skin, inducing patches, plaques and tumors. This clinical study showed that intratumoral injection of MV after systemic treatment by IFN-α (to avoid MV infection in IFN-α-sensitive healthy cells) induced local infection and a characteristic cytopathogenic effect of MV on tumor cells which was not abrogated by the presence of preexisting MV antibodies. Tumor regressions were observed in three patients with, interestingly, some regression of distant lesions where MV was not injected.

### 5.2. Phase I Clinical Trial on Chemotherapy-Refractory Ovarian Cancer

The oncolytic properties of MV have also been evaluated clinically in patients with Taxol- and platinum-refractory, recurrent ovarian cancers, who are seropositive for measles virus. In this phase I clinical study, Evanthia Galanis and colleagues (Mayo Clinic, Rochester, MN, USA) used MV-CEA, a modified Edmonston strain engineered to produce carcinoma embryonic antigen as a soluble maker, which allows the monitoring of MV replication by serum dosage [60]. Escalating doses were given to patients who were preimmunized against MV to assure the safety of the trial. Doses ranged from 10^3^ to 10^9^ TCID_50_ with no dose-limiting toxicity observed. Clinical objective responses were observed in fourteen of twenty-one patients, with a stabilization of disease for a median duration of 92.5 days. The stabilization of the disease was associated, in five patients, with a diminution of the tumor-specific marker, CA-125. The median survival time of the MV-treated patients (12.15 months) was double that of the expected median survival of the patient population (6 months). These results highlight the promising potential of oncolytic measles therapy in ovarian cancer patients.

### 5.3. Ongoing Clinical Trials

Three phase I clinical trials of antitumor virotherapy using MV are currently underway, recruiting patients with either recurrent ovarian cancer, mesothelioma or multiple myeloma. All of these trials are being performed at the Mayo Clinic, Rochester, Minnesota, USA, with a common outcome: to determine the maximum tolerated dose of a modified Edmonston strain of measles virus genetically engineered to produce the human thyroid sodium iodide symporter (MV-NIS) [95]. All recruited patients must be seropositive for measles virus with a level of serum anti-measles IgG greater than 20 EU/mL. The multiple myeloma clinical trial presents the particularity of evaluating the beneficial role of cyclophosphamide, an immunomodulatory agent able to suppress immune response, in MV-NIS-treated patients, to improve the spread of virus in tumor cells.

## 6. Conclusions

Antitumor virotherapy is a promising strategy to treat cancer. The broad range of cancer types sensitive to attenuated MV, the relatively high safety of attenuated MV for vaccinating children, the high immunogenicity of MV, and progress in industrial virus production, make MV a very good candidate for the development of this strategy. Furthermore, MV can be engineered to express transgenes to increase its oncolytic efficiency, to allow tracking of viral replication *in vivo,* or to increase its immune-stimulatory effects.
